# Outcomes of conversion surgery following chemotherapy for initially unresectable metastatic pancreatic ductal adenocarcinoma: a retrospective cohort study in Taiwan

**DOI:** 10.1007/s00432-025-06353-0

**Published:** 2025-10-30

**Authors:** Ping-Jui Su, Wei-Hsun Lu, Ting-Kai Liao, Chih-Jung Wang, Ying-Jui Chao, Yan-Shen Shan

**Affiliations:** 1 Division of General Surgery, Department of Surgery, National Cheng Kung University Hospital, College of Medicine, National Cheng Kung University, 138, Sheng-Li Road, Tainan, 70428 Taiwan, ROC; 2 Institute of Clinical Medicine, College of Medicine, National Cheng Kung University, Tainan, Taiwan, ROC

**Keywords:** Chemotherapy, Conversion surgery, Metastasis, Pancreatic ductal adenocarcinoma (PDAC), Survival, Unresectable

## Abstract

**Purpose:**

Pancreatic ductal adenocarcinoma (PDAC) is an aggressive cancer with a high mortality rate. For patients with metastatic PDAC (mPDAC) initially deemed unresectable, systemic chemotherapy followed by conversion surgery may offer an improvement in survival. This study aimed to compare survival between mPDAC patients undergoing conversion surgery versus chemotherapy alone, and identify factors associated with recurrence following conversion surgery.

**Methods:**

We conducted a retrospective cohort study of patients with mPDAC treated with systemic chemotherapy at National Cheng Kung University Hospital, Taiwan, between September 2020 and January 2023. Patients who subsequently underwent conversion surgery were analyzed to identify factors associated with recurrence. Clinicopathologic, treatment, and surgical variables were extracted from medical records. Recurrence-free survival (RFS) was defined from the date of conversion surgery to recurrence or death. Survival outcomes were estimated using the Kaplan–Meier method and compared with the log-rank test. Cox proportional hazards regression with stepwise selection was applied to identify independent predictors of recurrence.

**Results:**

Among 151 patients who underwent chemotherapy, 33 subsequently received conversion surgery. In the patients who received conversion surgery, male sex (HR 4.33, 95% CI 1.60–11.72), tumor location in the head/uncinate process (HR 2.79, 95% CI 1.03–7.58), and regression grade 2 (HR 4.65, 95% CI 1.41–15.30) were significantly associated with worse RFS.

**Conclusion:**

Among patients with mPDAC who underwent conversion surgery after chemotherapy, several factors were independently associated with shorter RFS, including male sex, tumor location in the pancreatic head/uncinate process, and histologic regression grade 2.

**Supplementary Information:**

The online version contains supplementary material available at 10.1007/s00432-025-06353-0.

## Introduction

Pancreatic ductal adenocarcinoma (PDAC) is one of the most aggressive and lethal cancers and the seventh leading cause of cancer-related mortality worldwide (Park et al. 2021). In 2020, there were about 496,000 new cases of PDAC and 466,000 deaths due to the disease globally. Despite advances in diagnosis and treatments, the overall 5-year survival rate for PDAC is only about 10% (Principe et al. 2021). This is largely attributed to diagnosis at a late stage of the disease, and metastasis at the time of diagnosis. Systemic chemotherapy is the standard treatment for patients with metastatic PDAC (mPDAC), but only offers a modest survival benefit.

In recent years, conversion surgery has gained attention as a potential option to extend survival in patients with mPDAC (Yanagimoto et al. 2020). This approach involves using systemic chemotherapy to downstage initially unresectable tumors to a resectable state. While conversion surgery has shown promising results in select patients, its success depends on various clinical and biological factors (Mataki et al. 2021). Identifying these factors is essential to optimizing patient selection and improving surgical outcomes.

Factors such as tumor biology, patient performance status, and chemotherapy treatment response influence the prognosis of mPDAC (Dell'Aquila et al. 2020; Yu et al. 2021). Important predictors of treatment outcomes include CA19-9 level, Response Evaluation Criteria in Solid Tumors (RECIST) characteristics, and tumor regression grade (Principe et al. 2021; van Veldhuisen et al. 2018). However, their specific roles in the context of conversion surgery require further evaluation. Additionally, perioperative factors such as resection margin status, lymph node involvement, and metastasectomy may impact recurrence-free survival (RFS); however, their importance remains underexplored (Blomstrand et al. 2023).

Although interest in conversion surgery for mPDAC is increasing, its clinical use remains limited due to unclear selection criteria, variable treatment responses, and inconsistent survival outcomes (Lingyu et al. 2023). Importantly, few studies, particularly in Asian populations, have systematically examined recurrence patterns or predictive factors, underscoring the need for real-world evidence to guide clinical decision-making. In this context, our major rationale was to identify clinicopathologic factors associated with recurrence after conversion surgery. By delineating predictors of recurrence, the ultimate goals are to improve patient selection, refine perioperative treatment strategies, and ultimately optimize outcomes in this challenging patient population.

Therefore, the purpose of this study was to identify factors associated with recurrence in patients who underwent conversion surgery. We hypothesized that specific demographic or clinicopathological features would predict recurrence following conversion surgery.

## Patients and methods

### Study design and patient selection

This retrospective cohort study reviewed the medical records of patients with mPDAC treated at a tertiary medical center, National Cheng Kung University Hospital (NCKUH), Taiwan. Inclusion criteria were: (1) Histologically confirmed mPDAC; (2) Malignancy considered unresectable at diagnosis; (3) Received initial systemic chemotherapy between September 2020 and January 2023; (4) Completion of at least one chemotherapy regimen; and (5) Availability of complete clinical and outcome data in the medical records. Exclusion criteria included patients lost to follow-up, with incomplete data, or who failed to complete a chemotherapy regimen. Subsequently, patients who received conversion surgery were divided into those with and without recurrence, to evaluate clinical factors associated with recurrence risk.

### Diagnosis and treatment algorithm

Diagnosis of unresectable mPDAC was based on clinical staging using contrast-enhanced abdominal and chest computed tomography (CT) scans and endoscopic ultrasound (EUS). Patients underwent repeat contrast-enhanced CT after approximately three months of systemic chemotherapy to assess treatment response and resectability. Conversion surgery was considered based on the following criteria: (1) Achieving a partial response (PR), stable disease (SD), or complete response (CR) after at least three months of chemotherapy, as evaluated by RECIST criteria; (2) Downstaging of the primary tumor to a resectable state, with a reasonable likelihood of achieving an R0 resection based on imaging; (3) No new metastases and stable or improved metastatic lesions sustained for more than six months. Patients with lung metastases or para-aortic lymph node (LN16) involvement were eligible for surgery if lesions were stable or improved, whereas liver metastases or peritoneal carcinomatosis required possibility of complete resection for surgical eligibility. According to our institutional protocol, all patients who underwent conversion surgery received an additional 6 months of systemic chemotherapy based on their preoperative regimen, followed by 1 year of maintenance oral chemotherapy.

### Data collection and endpoints

Demographic and clinical data were extracted from the medical records, including age, sex, tumor location, clinical staging (T and N stage), tumor size before and after treatment, number and sites of metastases, and Eastern Cooperative Oncology Group (ECOG) performance status. Laboratory data, such as CA19-9 levels before and after chemotherapy, were also recorded. Details of initial treatment included chemotherapy regimen, duration of treatment, and tumor response assessed using RECIST version 1.1 criteria. For patients who underwent conversion surgery, additional variables included surgical method, vascular resection, metastectomy, operative time, blood loss, and length of hospital stay. Pathological characteristics recorded were histological differentiation, lymph node status, resection margins, and tumor regression grade.

Recurrence was confirmed by follow-up imaging and/or clinical documentation. RFS was defined as the interval from the date of conversion surgery to the first occurrence of radiologically confirmed recurrence or death from any cause. All data were reviewed by two independent investigators to ensure accuracy and consistency.

### Statistical analysis

Categorical variables were presented as count (percentage) and compared using the chi-squared test or Fisher’s exact test, as appropriate. Continuous data with a normal distribution were presented as mean ± standard deviation (SD) and compared using the two-sample t-test. Data without a normal distribution were presented as median and interquartile range (IQR) and analyzed using the Wilcoxon Rank Sum test. The Shapiro–Wilk test was used to assess normality. Cases with missing data for any variables used in regression models were excluded from the respective analyses (complete-case analysis). For patients who underwent conversion surgery, multiple Cox proportional hazards regression was performed to identify factors associated with recurrence using a stepwise selection method. Variables with a *p* value < 0.25 were eligible for entry into the model, and those with *p* < 0.1 were retained in the final model. Stepwise selection was used to identify the most parsimonious set of predictors. The recurrence candidate factors included age, sex, tumor location, RECIST response, metastectomy, metastasis site (lung, liver, para-aortic lymph node, and peritoneal carcinomatosis), lymph node positivity after surgery, resection margin, and tumor regression grade. Time to event was calculated from the date of conversion surgery to the date of recurrence, death, or last follow-up. Patients without events were censored at their last known clinical follow-up. The Kaplan–Meier method was used to estimate RFS between groups, and survival curves were compared using the log-rank test. A two-sided *p* value < 0.05 was considered statistically significant. Data management and statistical analyses were conducted using SAS version 9.4 software (SAS Institute Inc., Cary, NC, USA).

## Results

### Patient characteristics and comparison by conversion surgery status

A total of 151 patients with unresectable mPDAC underwent initial chemotherapy between September 2020 and January 2023 at our hospital. Of the patients, 33 had conversion surgery. Patient characteristics are summarized in Table 1. The median patient age was 63 years, and 62% were male. Compared to patients who did not receive conversion surgery, those who received conversion surgery had smaller tumors, lower CA19-9 levels, longer follow-up duration, a lower proportion of advanced clinical stage disease (T4 and N2), liver metastasis, ECOG score 1–2, and death (Table 1).

**Table 1:** Patient characteristics

Variables	Total (N = 151)	Conversion surgery		*p* value
		Yes (n = 33)	No (n = 118)	
Sex, male	94 (62.3)	17 (51.5)	77 (65.3)	0.150
Age, years	63.3 (55.1–70.0)	62.8 (53.1–69.4)	63.4 (55.7–70.3)	0.471
Tumor location				
Head/uncinate process	58 (38.4)	10 (30.3)	48 (40.7)	0.279
Neck/body/tail	93 (61.6)	23 (69.7)	70 (59.3)	
Clinical T stage				
0	1 (0.7)	1 (3.0)	0 (0.0)	**< 0.001**
1	14 (9.3)	12 (36.4)	2 (1.7)	
2	32 (21.2)	11 (33.3)	21 (17.8)	
3	36 (23.8)	7 (21.2)	29 (24.6)	
4	68 (45.0)	2 (6.1)	66 (55.9)	
Clinical N stage				
0	26 (17.2)	19 (57.6)	7 (5.9)	**< 0.001**
1	53 (35.1)	11 (33.3)	42 (35.6)	
2	72 (47.7)	3 (9.1)	69 (58.5)	
Tumor size before treatment, mm	40 (30–52)	36 (23–45)	40 (32–55)	**0.027**
> 30	111 (73)	18 (54)	93 (79)	**0.005**
Number of metastatic sites				
1	121 (80.1)	32 (97.0)	89 (75.4)	**0.006**
2–3	30 (19.9)	1 (3.0)	29 (24.6)	
Metastatic sites				
Lung	22 (14.6)	6 (18.2)	16 (13.6)	0.577
Liver	98 (64.9)	15 (45.5)	83 (70.3)	**0.008**
Para-aortic lymph node	18 (11.9)	6 (18.2)	12 (10.2)	0.229
Peritoneal carcinomatosis	39 (25.8)	5 (15.2)	34 (28.8)	0.113
ECOG score				
0	20 (13.2)	9 (27.3)	11 (9.3)	**0.033**
1	128 (84.8)	24 (72.7)	104 (88.1)	
2	3 (2.0)	0 (0.0)	3 (2.5)	
CA19-9 before treatment, U/ml	929 (117–5167)	121 (44–1207)	1230 (185–6529)	**0.001**
< 37	23 (15.2)	7 (21.2)	16 (13.6)	**0.001**
37–400	36 (23.8)	15 (45.5)	21 (17.8)	
> 400	92 (60.9)	11 (33.3)	81 (68.6)	
Death	129 (85.4)	17 (51.5)	112 (94.9)	**< 0.001**
Follow-up duration, month	12.2 (6.0–22.1)	23.4 (16.9–31.0)	9.9 (5.1–15.8)	**< 0.001**

Categorical variables are presented as count (percentage), and continuous data with a normal distribution are presented as mean ± standard deviation (SD); otherwise data are presented as the median and interquartile range (IQR)

Significant values (p < 0.05) are shown in bold

ECOG: Eastern Cooperative Oncology Group

### Recurrence after conversion surgery

**Fig. 1:**
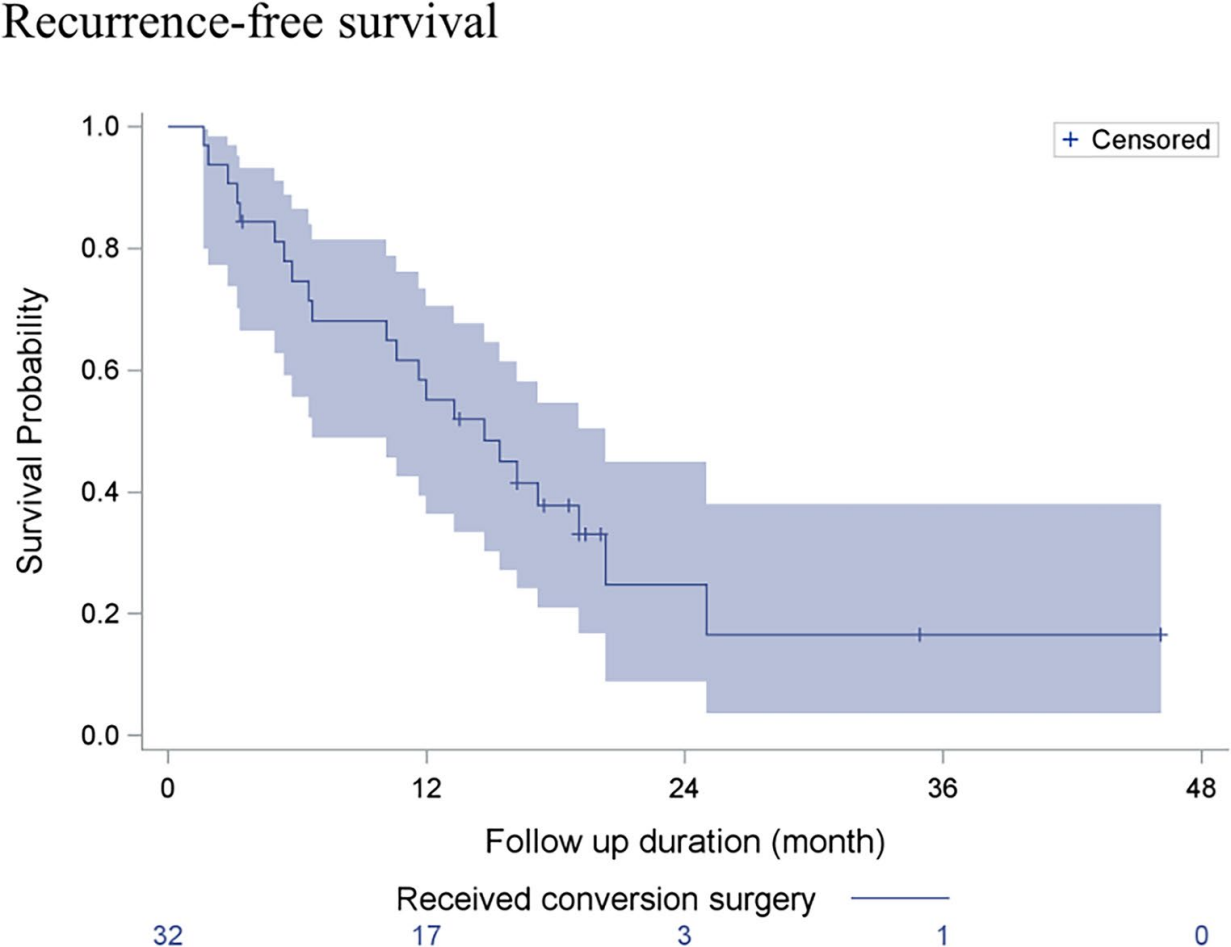
Recurrence-free survival (RFS) curve of patients who received conversion surgery. The median RFS was 13.54 months

Figure 1 depicts the Kaplan–Meier curve of RFS in patients who underwent conversion surgery. The median RFS after conversion surgery was 13.54 months (Fig. 1). The characteristics of patients who received conversion surgery and had a recurrence and those who did not were compared. Patients with and without recurrence had similar demographic and clinical characteristics, initial treatment, and perioperative period (Table 2).

**Table 2:** Characteristics of patients who received conversion surgery categorized by the presence of recurrence

Study variable	Total (n = 33)	Recurrence		*p* value
		Yes (n = 23)	No (n = 10)	
Sex, male	17 (51.5)	14 (60.9)	3 (30.0)	0.141
Age, year	60.6 ± 10.6	61.7 ± 10.0	58.0 ± 12.0	0.363
Tumor location				
Head/uncinate process	10 (30.3)	8 (34.8)	2 (20.0)	0.682
Neck/body/tail	23 (69.7)	15 (65.2)	8 (80.0)	
Clinical T stage				
0–2	24 (72.7)	16 (69.6)	8 (80.0)	0.686
3–4	9 (27.3)	7 (30.4)	2 (20.0)	
Clinical N stage				
0	19 (57.6)	12 (52.2)	7 (70.0)	0.455
1–2	14 (42.4)	11 (47.8)	3 (30.0)	
Tumor size before treatment, mm	36.4 ± 15.4	39.2 ± 15.3	30.1 ± 14.4	0.120
> 30	18 (54.5)	14 (60.9)	4 (40.0)	0.448
Number of metastatic sites				
1	32 (97.0)	22 (95.7)	10 (100.0)	> 0.999
2–3	1 (3.0)	1 (4.3)	0 (0.0)	
Metastatic sites				
Lung	6 (18.2)	5 (21.7)	1 (10.0)	0.640
Liver	15 (45.5)	9 (39.1)	6 (60.0)	0.448
Para-aortic lymph node	6 (18.2)	4 (17.4)	2 (20.0)	> 0.999
Peritoneal carcinomatosis	5 (15.2)	4 (17.4)	1 (10.0)	> 0.999
ECOG score				
0	9 (27.3)	7 (30.4)	2 (20.0)	0.686
1	24 (72.7)	16 (69.6)	8 (80.0)	
CA19-9 before treatment, U/ml	121 (44–1,207)	177 (49–1,240)	75.8 (35–188)	0.136
< 37	7 (21.2)	3 (13.0)	4 (40.0)	0.286
37–400	15 (45.5)	11 (47.8)	4 (40.0)	
> 400	11 (33.3)	9 (39.1)	2 (20.0)	
Initial Treatment				
Regimen				
SLOG	7 (21.2)	5 (21.7)	2 (20.0)	0.953
AG	11 (33.3)	7 (30.4)	4 (40.0)	
AGSL	9 (27.3)	7 (30.4)	2 (20.0)	
AGC	6 (18.2)	4 (17.4)	2 (20.0)	
Duration of treatment to operation, months	5.8 ± 2.4	6.2 ± 2.5	4.8 ± 2.0	0.122
< 3	2 (6.1)	0 (0.0)	2 (20.0)	0.086
3–6	15 (45.5)	10 (43.5)	5 (50.0)	
> 6	16 (48.5)	13 (56.5)	3 (30.0)	
RECIST				
SD	10 (31.3)	8 (36.4)	2 (20.0)	0.440
PR	22 (68.8)	14 (63.6)	8 (80.0)	
Missing	1	1	0	
Tumor size after treatment, mm	24.7 ± 14.2	26.9 ± 15.5	19.7 ± 9.5	0.185
CA19-9 months after treatment, U/ml	28.9 (10.4–88.8)	30.5 (13.5–88.8)	26.2 (6.1–95.0)	0.710
< 3 months	19 (57.6)	13 (56.5)	6 (60.0)	0.565
3–6 months	10 (30.3)	8 (34.8)	2 (20.0)	
6–9 months	4 (12.1)	2 (8.7)	2 (20.0)	
Perioperative				
Surgical method				
Distal pancreatectomy	18 (54.5)	11 (47.8)	7 (70.0)	0.701
Extended distal pancreatectomy	3 (9.1)	2 (8.7)	1 (10.0)	
Pancreaticoduodenectomy	10 (30.3)	8 (34.8)	2 (20.0)	
Total pancreatectomy	2 (6.1)	2 (8.7)	0 (0.0)	
Vascular resection				
No	21 (63.6)	15 (65.2)	6 (60.0)	0.859
SMV	8 (24.2)	5 (21.7)	3 (30.0)	
SMA/CA	4 (12.1)	3 (13.0)	1 (10.0)	
Metastasectomy				
No	26 (78.8)	19 (82.6)	7 (70.0)	0.646
Yes	7 (21.2)	4 (17.4)	3 (30.0)	
Operation time, min	327.7 ± 112.1	344.3 ± 115.3	289.6 ± 99.2	0.203
Blood loss, ml	350 (200–700)	350 (200–700)	325 (150–800)	0.622
Postoperative hospital stays, days	16.8 ± 8.6	18.3 ± 9.5	13.5 ± 4.6	0.060
Pathological characteristics after conversion surgery				
Histology differentiation				
Moderate	28 (87.5)	19 (86.4)	9 (90.0)	> 0.999
Poor	4 (12.5)	3 (13.6)	1 (10.0)	
Missing	1	1	0	
LN positive	14 (42.4)	11 (47.8)	3 (30.0)	0.455
LN harvested number	20.4 ± 8.3	20.6 ± 8.1	20.1 ± 9.2	0.885
Resection margin				
R0	27 (81.8)	18 (78.3)	9 (90.0)	0.640
R1–R2	6 (18.2)	5 (21.7)	1 (10.0)	
R2	1 (3.0)	0 (0.0)	1 (10.0)	
Tumor regression grade				
Higher (2)	24 (72.7)	18 (78.3)	6 (60.0)	0.400
Lower (0–1)	9 (27.3)	5 (21.7)	4 (40.0)	
Time to recurrence or last visit, months	13.3 ± 9.8	10.0 ± 6.8	20.9 ± 11.7	

Categorical variables are presented as count (percentage), and continuous data with a normal distribution are presented as mean ± standard deviation (SD); otherwise data are presented as the median and interquartile range (IQR)

Chemotherapy regimens: SLOG: TS-1, Leucovorin, Oxaliplatin, Gemcitabine; AG: Nab-paclitaxel, Gemcitabine; AGSL: Nab-paclitaxel, Gemcitabine, TS-1, Leucovorin; AGC: Nab-paclitaxel, Gemcitabine, Cisplatin

RECIST: Response Evaluation Criteria in Solid Tumors; PR: partial response; SD: stable disease; ECOG: Eastern Cooperative Oncology Group; LN: lymph node; OP: operation; SMV: superior mesenteric vein; SMA/CA: superior mesenteric artery/celiac axis

**Fig. 2:**
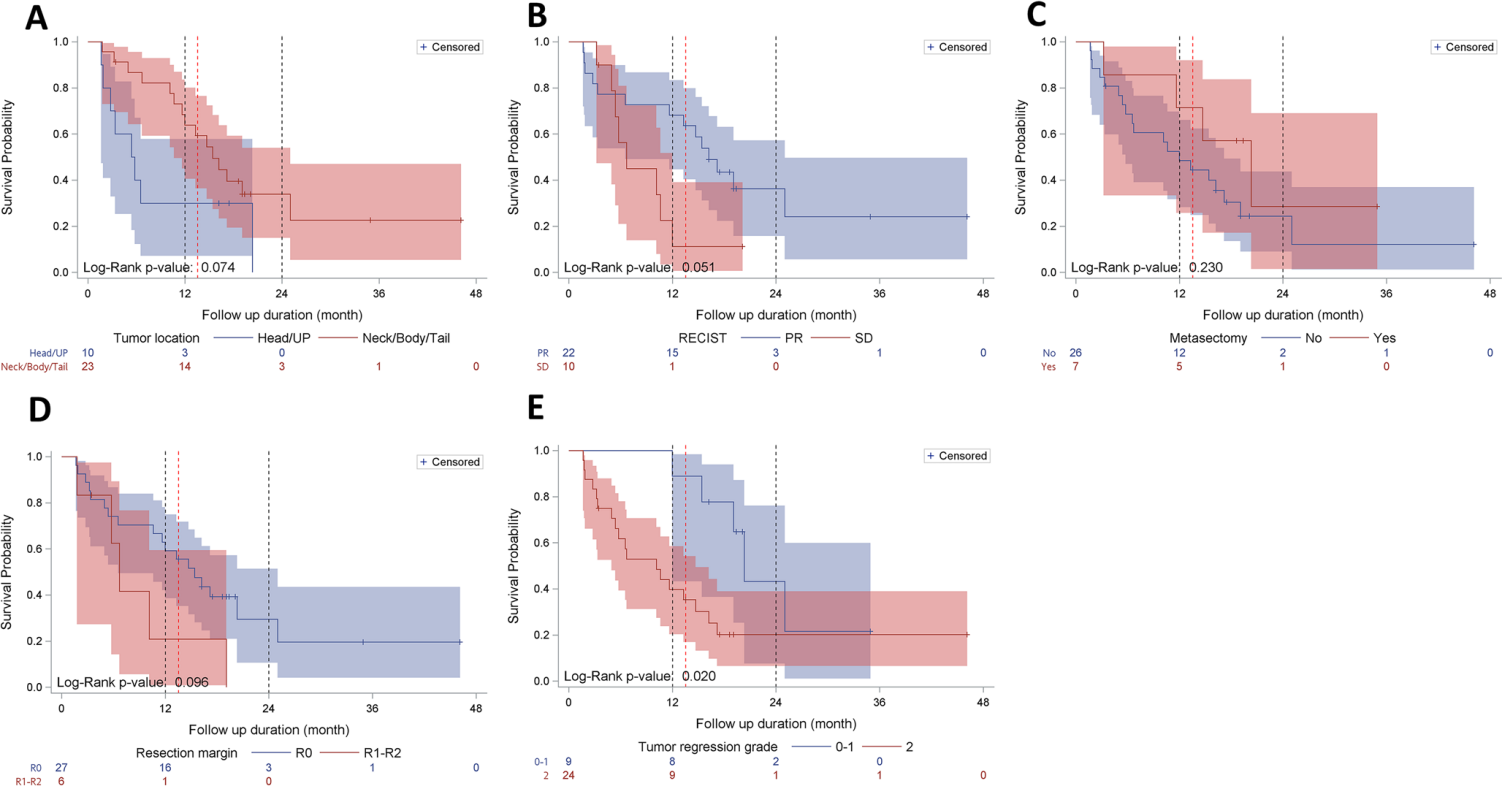
Recurrence-free survival (RFS) curves of patient who received conversion surgery stratified by **A** Tumor location: head versus neck/body/tail pancreas; **B** Response Evaluation Criteria in Solid Tumors (RECIST): partial response (PR) versus stable disease (SD); **C** Metastectomy: yes versus no; and **D** Resection margins: R0 versus R1–R2. **E** Tumor regression grade: 0–1 versus 2. The red dashed line represents the median RFS (13.54 months), while the black dashed lines indicate the reference lines for 1-year and 2-year survival

The Kaplan–Meier curves for RFS stratified by tumor location (head/uncinate process vs. neck/body/tail), RECIST response (PR vs. SD), metastasectomy (yes vs. no), resection margin (R0 vs. R1–R2), and tumor regression grade (0–1 vs. 2) are presented in Fig. 2 and were compared using the log-rank test (Fig. 2). Specifically, patients with tumor regression grade 0–1 had a higher RFS than those with tumor regression grade 2 (*p* = 0.020, Fig. 2E).

Supplementary Tables 1–5 illustrate survival probabilities across various clinical subgroups.

### Factors associated with recurrence after conversion surgery

As shown in Table 3, the factors significantly associated with recurrence in patients who received conversion surgery were male sex, tumor location, RECIST, and tumor regression grade. Male patients had a significantly higher risk of recurrence compared to females [hazard ratio (HR) = 4.33, 95% confidence interval (CI) = 1.60–11.72, *p* = 0.004]. Compared to tumors in the neck/body/tail of the pancreas, those located in the head/uncinate process were associated with a higher risk of recurrence (HR 2.79, 95% CI 1.03–7.58, *p* = 0.044). Stable disease by RECIST criteria was associated with a higher risk of recurrence compared to a partial response (HR 4.08, 95% CI 1.39–12.0, *p* = 0.011). Tumors with regression grade 2 were associated with a significantly higher risk of recurrence compared to grades 0–1 (HR 4.65, 95% CI 1.41–15.30, *p* = 0.012) (Table 3).

**Table 3:** Factors associated with recurrence

Variables	HR (95% CI)	*p* value
Sex		**0.004**
Male	4.33 (1.60–11.72)	
Female	Ref	
Tumor location		**0.044**
Head/uncinate process	2.79 (1.03–7.58)	
Neck/body/tail	Ref	
RECIST		**0.011**
SD	4.08 (1.39–12.00)	
PR	Ref	
Tumor regression grade		**0.012**
Higher (2)	4.65 (1.41–15.30)	
Lower (0–1)	Ref	

Significant values (p < 0.05) are shown in bold

PR: Partial response; SD: stable disease; HR: hazard ratio; CI: confidence interval; RECIST: Response Evaluation Criteria in Solid Tumors

## Discussion

This retrospective analysis of patients with initially unresectable mPDAC treated at a single center in Taiwan provides key insights into the potential role of conversion surgery following systemic chemotherapy. Specifically, we identified four independent factors associated with recurrence after conversion surgery: male sex, tumor location in the pancreatic head or uncinate process, SD by RECIST criteria (as compared to partial response), and higher tumor regression grade. This finding contribute to the growing body of evidence supporting the use of conversion surgery in appropriately selected mPDAC patients, while also highlighting clinical and pathological features that may predict postoperative recurrence.

Pancreatic cancer is one of the most aggressive malignancies, and the survival rate is low, in part, because early signs and symptoms are non-specific and the disease is usually diagnosed at an advanced stage (Garajova et al. 2023; Hank & Strobel 2019). Surgery followed by chemotherapy is the standard treatment for pancreatic cancer that is considered resectable at diagnosis, and there are no high-risk factors (Garajova et al. 2023). In the past, treatment options for unresectable pancreatic cancer were limited, with systemic chemotherapy primarily used for palliation rather than for potential downstaging. However, with the advent of more effective chemotherapy regimens, select patients may experience significant tumor regression, potentially allowing for conversion surgery. However, this approach remains investigational and is not yet the standard of care (Garajova et al. 2023; Hank & Strobel 2019; Klaiber & Hackert 2019; Y. N. Lee et al. 2024).

A recently published meta-analysis reported that conversion surgery significantly improved survival (HR 0.55) in patients with initially unresectable pancreatic cancer who had a favorable response to chemotherapy (Zhou et al. 2022). The two most common chemotherapy regimens for unresectable pancreatic cancer are FOLFIRINOX or gemcitabine plus nab-paclitaxel (Ide et al.). Ide et al*.* (Ide et al.) recently reviewed the records of 318 patients with unresectable pancreatic cancer initially treated with one of the aforementioned chemotherapy regimens. OS was significantly longer in patients who received conversion surgery than in those who did not (median 33 vs. 11 months; a HR 0.32). The median survival after conversion surgery was 44 months in patients with initially locally advanced disease and 21 months in patients with metastasis at diagnosis. Notably, factors that were associated with the opportunity for conversion surgery were locally advanced disease, no liver metastasis, CA19-9 ≤ 37, and an adequate response to chemotherapy (Ide et al. 2023).

In a study published in 2024, Higashi et al. (2024) examined predictors for attaining the requirements for conversion surgery in patients with unresectable PDAC. The analysis showed that total protein (HR 2.81), neutrophil-to-lymphocyte ratio (HR 0.53), and lymphocyte-to-monocyte ratio (HR 1.28) were significantly associated with conversion surgery. These findings emphasize the emerging role of conversion surgery and the importance of related biomarkers and clinical indicators.

In our study, conversion surgery was considered only for patients with no new metastases, radiologically stable or improved metastatic lesions sustained for at least six months, and a primary tumor downstaged to a resectable state with the potential for R0 resection. By contrast, the Heidelberg group (Hank et al. 2023) applied more flexible criteria, selecting patients based on RECIST-defined radiological response and concurrent declines in CA19-9 and CEA, and typically proceeding to surgery after six cycles of chemotherapy, often within 5–9 months of diagnosis. Thus, our approach was more conservative, while theirs relied on integrated radiological and biological response to guide earlier surgical intervention. Future research are warranted to directly compare different selection criteria to determine their relative impact on outcomes.

Our analysis identified several key prognostic factors for patients undergoing conversion surgery. Other studies also examined conversion surgery outcomes and prognostic factors influencing post-surgical survival. For example, Satoi et al. (2020) reported that patients who received conversion surgery based on clinical response to chemotherapy and decreased CA19-9 level had a median survival time of 36 months. The multivariate analysis indicated that a decreased CA19-9 level was a predictor of resectability, OS, and disease-free survival (DFS). Yanagimoto et al*.* (Yanagimoto et al. 2020) reported that the median survival time and 5-year survival of patients who received conversion surgery were 37 months and 34%, compared to 9 months and 1% for patients who did not have surgery. Notably, lymph node metastasis, positive washing cytology, large tumor size (> 35 mm), and no postoperative adjuvant chemotherapy were statistically significant predictors of early recurrence, and the site of pancreatic lesion and administration of postoperative adjuvant chemotherapy were statistically significant prognostic factors for OS. In another study that included 638 patients, the median OS of patients with conversion surgery, resectable cancer, borderline resectable, unresectable locally advanced, and unresectable and distant metastases was 74 months, 33 months, 23 months, 16 months, and 9 months, respectively. The multivariate analysis indicated that the presence or absence of chemoradiotherapy and a RECIST PR or CR of the primary tumor were statistically significant prognostic factors for OS in patients who received conversion surgery (Mataki et al. 2021). In another recent study, Hank et al. (2023) reported that in patients with a complete pathological response of metastasis, the OS was 26 months, compared to 8 months in patients who did not have surgery. As seen in other studies, CA19-9 level was an independent prognostic factor for all patients in the study.

Other studies have also reported similar findings. Notably, studies reported that a decrease in CA19-9 level was a positive prognostic factor (Igarashi et al. 2023; Lee et al. 2023; Li et al. 2023). In a recent study that included 67 patients with unresectable pancreatic cancer, Li et al. (2023) reported that R0 resection and a decrease in the levels of tumor markers after conversion surgery were the most important prognostic factors for PFS. Lee et al*.* (Lee et al. 2023) reported that a CA19-9 level > 200 U/ml after neoadjuvant chemotherapy was an independent factor for early recurrence after conversion surgery (HR 7.2). Igarashi et al. (2023) retrospectively analyzed the records of 144 patients with unresectable locally advanced PDAC. In patients who received induction gemcitabine and nab-paclitaxel followed by additional chemoradiation therapy, the multivariate analysis revealed CA19-9 normalization prior to surgery (HR 0.23) and a prognostic nutritional index ≥ 42 (HR 0.05) were independent prognostic factors for prolonged survival.

Overall, the identification of prognostic factors in this study contributes to the growing body of literature on the treatment of PDAC. These findings may help guide the implementation of tailored postoperative or adjuvant therapies aimed at reducing recurrence risk and improving long-term outcomes.

### Strengths and limitations

The strengths of this study include its focus on a patient population with unresectable mPDAC undergoing conversion surgery, which is a relatively underexplored topic of research. The data collection was comprehensive, including clinical, pathological, and outcome data, allowing for a detailed analysis of factors influencing survival and recurrence. Despite these strengths, the study has several limitations that should be considered. First, the retrospective design introduces inherent biases, particularly related to patient selection. The single-center nature of the study might limit the generalizability of the findings to other settings or populations due to differences in treatment protocols and patient demographics. Furthermore, the small sample size, especially for patients who underwent conversion surgery, may affect the statistical power to detect significant differences and interactions between various factors. Another limitation is the lack of molecular or genetic data, which could provide deeper insights into the biological behavior of tumors and possibly explain some of the observed variations in treatment response and survival outcomes.

## Conclusion

This study identified several factors associated with recurrence after conversion surgery among patients with mPDAC, including male sex and tumor location in the pancreatic head/uncinate process, stable disease by RECIST criteria (versus partial response), and higher tumor regression grade. These findings may help identify patients at higher risk of recurrence who could benefit from intensified surveillance and focused postoperative management.

## Supplementary Information

Below is the link to the electronic supplementary material.

Supplementary Material 1

## Data Availability

No datasets were generated or analysed during the current study.
