# Supplementary material for: Outcomes of conversion surgery following chemotherapy for initially unresectable metastatic pancreatic ductal adenocarcinoma: a retrospective cohort study in Taiwan
Source: J Cancer Res Clin Oncol. 2025 Oct 30;151(12):308. doi: 10.1007/s00432-025-06353-0 (PMC12575905; doi:10.1007/s00432-025-06353-0)
Supplement: Supplementary file 1 — Supplementary Material 1 [file 432_2025_6353_MOESM1_ESM.docx]

**Supplemental Table 1. The life table of Tumor location**

| *Tumor location* | *Month* | *Survival Probability* | *SDF_LCL* | *SDF_UCL* | *At Risk* |
| --- | --- | --- | --- | --- | --- |
| Head/UP | 0.0000 | 1.00000 | 1.00000 | 1.00000 | 10 |
| Head/UP | 1.6393 | 0.90000 | 0.47301 | 0.98528 | 10 |
| Head/UP | 1.8689 | 0.80000 | 0.40869 | 0.94587 | 9 |
| Head/UP | 2.7869 | 0.70000 | 0.32872 | 0.89195 | 8 |
| Head/UP | 3.3443 | 0.60000 | 0.25267 | 0.82722 | 7 |
| Head/UP | 5.3770 | 0.50000 | 0.18361 | 0.75317 | 6 |
| Head/UP | 5.7705 | 0.40000 | 0.12269 | 0.67020 | 5 |
| Head/UP | 6.5246 | 0.30000 | 0.07113 | 0.57787 | 4 |
| Head/UP | 20.3279 | 0.00000 | 0.00000 | 0.00000 | 1 |
| Neck/Body/Tail | 0.0000 | 1.00000 | 1.00000 | 1.00000 | 23 |
| Neck/Body/Tail | 1.7705 | 0.95652 | 0.72934 | 0.99376 | 23 |
| Neck/Body/Tail | 3.2131 | 0.91304 | 0.69495 | 0.97752 | 22 |
| Neck/Body/Tail | 4.9508 | 0.86739 | 0.64271 | 0.95525 | 20 |
| Neck/Body/Tail | 6.6885 | 0.82174 | 0.59173 | 0.92917 | 19 |
| Neck/Body/Tail | 10.1311 | 0.77609 | 0.54261 | 0.90023 | 18 |
| Neck/Body/Tail | 10.6230 | 0.73043 | 0.49534 | 0.86897 | 17 |
| Neck/Body/Tail | 11.6393 | 0.68478 | 0.44981 | 0.83572 | 16 |
| Neck/Body/Tail | 11.9672 | 0.63913 | 0.40594 | 0.80070 | 15 |
| Neck/Body/Tail | 13.2787 | 0.59348 | 0.36364 | 0.76406 | 14 |
| Neck/Body/Tail | 14.6885 | 0.54402 | 0.31781 | 0.72376 | 12 |
| Neck/Body/Tail | 15.4098 | 0.49457 | 0.27433 | 0.68164 | 11 |
| Neck/Body/Tail | 16.1967 | 0.44511 | 0.23308 | 0.63771 | 10 |
| Neck/Body/Tail | 17.1803 | 0.39565 | 0.19404 | 0.59195 | 9 |
| Neck/Body/Tail | 19.0820 | 0.33913 | 0.14970 | 0.54024 | 7 |
| Neck/Body/Tail | 25.0164 | 0.22609 | 0.05372 | 0.46951 | 3 |
| Neck/Body/Tail | 46.0984 | 0.22609 | 0.05372 | 0.46951 | 1 |
| Note: SDF_LCL/SDF_UCL, a variable that contains the lower/upper limits of the pointwise confidence intervals for the survival function. | | | | | |

**Supplemental Table 2. The life table of RECIST**

| *RECIST* | *Month* | *Survival Probability* | *SDF_LCL* | *SDF_UCL* | *At Risk* |
| --- | --- | --- | --- | --- | --- |
| PR | 0.0000 | 1.00000 | 1.00000 | 1.00000 | 22 |
| PR | 1.6393 | 0.95455 | 0.71870 | 0.99347 | 22 |
| PR | 1.7705 | 0.90909 | 0.68298 | 0.97646 | 21 |
| PR | 1.8689 | 0.86364 | 0.63444 | 0.95386 | 20 |
| PR | 2.7869 | 0.81818 | 0.58535 | 0.92757 | 19 |
| PR | 3.3443 | 0.77273 | 0.53743 | 0.89848 | 18 |
| PR | 6.5246 | 0.72727 | 0.49103 | 0.86711 | 17 |
| PR | 11.6393 | 0.68182 | 0.44619 | 0.83380 | 16 |
| PR | 13.2787 | 0.63636 | 0.40286 | 0.79875 | 15 |
| PR | 14.6885 | 0.58741 | 0.35615 | 0.76020 | 13 |
| PR | 15.4098 | 0.53846 | 0.31170 | 0.71983 | 12 |
| PR | 16.1967 | 0.48951 | 0.26938 | 0.67770 | 11 |
| PR | 17.1803 | 0.43512 | 0.22257 | 0.63074 | 9 |
| PR | 19.0820 | 0.36260 | 0.15763 | 0.57290 | 6 |
| PR | 25.0164 | 0.24173 | 0.05604 | 0.49677 | 3 |
| PR | 46.0984 | 0.24173 | 0.05604 | 0.49677 | 1 |
| SD | 0.0000 | 1.00000 | 1.00000 | 1.00000 | 10 |
| SD | 3.2131 | 0.90000 | 0.47301 | 0.98528 | 10 |
| SD | 4.9508 | 0.78750 | 0.38088 | 0.94259 | 8 |
| SD | 5.3770 | 0.67500 | 0.29059 | 0.88250 | 7 |
| SD | 5.7705 | 0.56250 | 0.20942 | 0.80917 | 6 |
| SD | 6.6885 | 0.45000 | 0.13877 | 0.72408 | 5 |
| SD | 10.1311 | 0.33750 | 0.07971 | 0.62723 | 4 |
| SD | 10.6230 | 0.22500 | 0.03422 | 0.51721 | 3 |
| SD | 11.9672 | 0.11250 | 0.00620 | 0.39102 | 2 |
| SD | 20.0984 | 0.11250 | 0.00620 | 0.39102 | 1 |

Note: SDF_LCL/SDF_UCL, a variable that contains the lower/upper limits of the pointwise confidence intervals for the survival function.

|  |
| --- |

**Supplemental Table 3. The life table of Metasectomy**

| *Metasectomy* | *Month* | *Survival Probability* | *SDF_LCL* | *SDF_UCL* | *At Risk* |
| --- | --- | --- | --- | --- | --- |
| No | 0.0000 | 1.00000 | 1.00000 | 1.00000 | 26 |
| No | 1.6393 | 0.96154 | 0.75694 | 0.99449 | 26 |
| No | 1.7705 | 0.92308 | 0.72603 | 0.98019 | 25 |
| No | 1.8689 | 0.88462 | 0.68358 | 0.96126 | 24 |
| No | 2.7869 | 0.84615 | 0.64043 | 0.93929 | 23 |
| No | 3.3443 | 0.80769 | 0.59811 | 0.91508 | 22 |
| No | 4.9508 | 0.76731 | 0.55349 | 0.88816 | 20 |
| No | 5.3770 | 0.72692 | 0.51064 | 0.85955 | 19 |
| No | 5.7705 | 0.68654 | 0.46933 | 0.82946 | 18 |
| No | 6.5246 | 0.64615 | 0.42941 | 0.79803 | 17 |
| No | 6.6885 | 0.60577 | 0.39078 | 0.76536 | 16 |
| No | 10.1311 | 0.56538 | 0.35338 | 0.73153 | 15 |
| No | 10.6230 | 0.52500 | 0.31718 | 0.69658 | 14 |
| No | 11.9672 | 0.48462 | 0.28215 | 0.66052 | 13 |
| No | 13.2787 | 0.44423 | 0.24831 | 0.62337 | 12 |
| No | 15.4098 | 0.39981 | 0.21105 | 0.58259 | 10 |
| No | 16.1967 | 0.35538 | 0.17578 | 0.54029 | 9 |
| No | 17.1803 | 0.30462 | 0.13583 | 0.49273 | 7 |
| No | 19.0820 | 0.24369 | 0.08962 | 0.43764 | 5 |
| No | 25.0164 | 0.12185 | 0.01160 | 0.36998 | 2 |
| No | 46.0984 | 0.12185 | 0.01160 | 0.36998 | 1 |
| Yes | 0.0000 | 1.00000 | 1.00000 | 1.00000 | 7 |
| Yes | 3.2131 | 0.85714 | 0.33405 | 0.97856 | 7 |
| Yes | 11.6393 | 0.71429 | 0.25815 | 0.91980 | 6 |
| Yes | 14.6885 | 0.57143 | 0.17187 | 0.83708 | 5 |
| Yes | 20.3279 | 0.28571 | 0.01442 | 0.69060 | 2 |
| Yes | 34.9180 | 0.28571 | 0.01442 | 0.69060 | 1 |

Note: SDF_LCL/SDF_UCL, a variable that contains the lower/upper limits of the pointwise confidence intervals for the survival function.

|  |
| --- |

**Supplemental Table 4. The life table of Resection margin**

| *Resection margin* | *Month* | *Survival Probability* | *SDF_LCL* | *SDF_UCL* | *At Risk* |
| --- | --- | --- | --- | --- | --- |
| R0 | 0.0000 | 1.00000 | 1.00000 | 1.00000 | 27 |
| R0 | 1.6393 | 0.96296 | 0.76494 | 0.99470 | 27 |
| R0 | 1.8689 | 0.92593 | 0.73504 | 0.98094 | 26 |
| R0 | 2.7869 | 0.88889 | 0.69390 | 0.96275 | 25 |
| R0 | 3.2131 | 0.85185 | 0.65203 | 0.94166 | 24 |
| R0 | 3.3443 | 0.81481 | 0.61093 | 0.91841 | 23 |
| R0 | 4.9508 | 0.77778 | 0.57088 | 0.89345 | 22 |
| R0 | 5.3770 | 0.74074 | 0.53192 | 0.86704 | 21 |
| R0 | 6.5246 | 0.70370 | 0.49402 | 0.83937 | 20 |
| R0 | 10.6230 | 0.66667 | 0.45714 | 0.81056 | 19 |
| R0 | 11.6393 | 0.62963 | 0.42123 | 0.78072 | 18 |
| R0 | 11.9672 | 0.59259 | 0.38626 | 0.74990 | 17 |
| R0 | 13.2787 | 0.55556 | 0.35222 | 0.71814 | 16 |
| R0 | 14.6885 | 0.51587 | 0.31571 | 0.68387 | 14 |
| R0 | 15.4098 | 0.47619 | 0.28052 | 0.64852 | 13 |
| R0 | 16.1967 | 0.43651 | 0.24663 | 0.61209 | 12 |
| R0 | 17.1803 | 0.39286 | 0.20943 | 0.57214 | 10 |
| R0 | 20.3279 | 0.29464 | 0.10605 | 0.51402 | 4 |
| R0 | 25.0164 | 0.19643 | 0.04147 | 0.43512 | 3 |
| R0 | 46.0984 | 0.19643 | 0.04147 | 0.43512 | 1 |
| R1-R2 | 0.0000 | 1.00000 | 1.00000 | 1.00000 | 6 |
| R1-R2 | 1.7705 | 0.83333 | 0.27312 | 0.97471 | 6 |
| R1-R2 | 5.7705 | 0.62500 | 0.14185 | 0.89305 | 4 |
| R1-R2 | 6.6885 | 0.41667 | 0.05599 | 0.76652 | 3 |
| R1-R2 | 10.1311 | 0.20833 | 0.00874 | 0.59506 | 2 |
| R1-R2 | 19.0820 | 0.00000 | 0.00000 | 0.00000 | 1 |

Note: SDF_LCL/SDF_UCL, a variable that contains the lower/upper limits of the pointwise confidence intervals for the survival function.

|  |
| --- |

**Supplemental Table 5. The life table of** **Tumor regression grade**

| *Tumor regression grade* | *Month* | *Survival Probability* | *SDF_LCL* | *SDF_UCL* | *At Risk* |
| --- | --- | --- | --- | --- | --- |
| 0-1 | 0.0000 | 1.00000 | 1.00000 | 1.00000 | 9 |
| 0-1 | 11.9672 | 0.88889 | 0.43297 | 0.98356 | 9 |
| 0-1 | 15.4098 | 0.77778 | 0.36475 | 0.93930 | 8 |
| 0-1 | 19.0820 | 0.64815 | 0.25317 | 0.87207 | 6 |
| 0-1 | 20.3279 | 0.43210 | 0.07553 | 0.76142 | 3 |
| 0-1 | 25.0164 | 0.21605 | 0.01029 | 0.59869 | 2 |
| 0-1 | 34.9180 | 0.21605 | 0.01029 | 0.59869 | 1 |
| 2 | 0.0000 | 1.00000 | 1.00000 | 1.00000 | 24 |
| 2 | 1.6393 | 0.95833 | 0.73921 | 0.99402 | 24 |
| 2 | 1.7705 | 0.91667 | 0.70606 | 0.97848 | 23 |
| 2 | 1.8689 | 0.87500 | 0.66076 | 0.95788 | 22 |
| 2 | 2.7869 | 0.83333 | 0.61481 | 0.93395 | 21 |
| 2 | 3.2131 | 0.79167 | 0.56985 | 0.90752 | 20 |
| 2 | 3.3443 | 0.75000 | 0.52618 | 0.87907 | 19 |
| 2 | 4.9508 | 0.70588 | 0.47986 | 0.84770 | 17 |
| 2 | 5.3770 | 0.66176 | 0.43550 | 0.81461 | 16 |
| 2 | 5.7705 | 0.61765 | 0.39290 | 0.77996 | 15 |
| 2 | 6.5246 | 0.57353 | 0.35190 | 0.74384 | 14 |
| 2 | 6.6885 | 0.52941 | 0.31243 | 0.70632 | 13 |
| 2 | 10.1311 | 0.48529 | 0.27447 | 0.66745 | 12 |
| 2 | 10.6230 | 0.44118 | 0.23799 | 0.62721 | 11 |
| 2 | 11.6393 | 0.39706 | 0.20304 | 0.58560 | 10 |
| 2 | 13.2787 | 0.35294 | 0.16968 | 0.54255 | 9 |
| 2 | 14.6885 | 0.30252 | 0.13161 | 0.49416 | 7 |
| 2 | 16.1967 | 0.25210 | 0.09694 | 0.44326 | 6 |
| 2 | 17.1803 | 0.20168 | 0.06593 | 0.38957 | 5 |
| 2 | 46.0984 | 0.20168 | 0.06593 | 0.38957 | 1 |

Note: SDF_LCL/SDF_UCL, a variable that contains the lower/upper limits of the pointwise confidence intervals for the survival function.
